# Implementation and Analysis of ISM 2.4 GHz Wireless Sensor Network Systems in Judo Training Venues

**DOI:** 10.3390/s16081247

**Published:** 2016-08-06

**Authors:** Peio Lopez-Iturri, Erik Aguirre, Leyre Azpilicueta, José Javier Astrain, Jesús Villadangos, Francisco Falcone

**Affiliations:** 1 Electrical and Electronic Engineering Department, Public University of Navarre, Pamplona 31006, Spain; peio.lopez@unavarra.es (P.L.-I.); erik.aguirre@unavarra.es (E.A.); 2 School of Engineering and Sciences, Tecnologico de Monterrey, Campus Monterrey, Monterrey, NL 64849, Mexico; leyre.azpilicueta@itesm.mx; 3 Mathematical Engineering and Computer Science Department, Institute for Smart Cities, Public University of Navarre, Pamplona 31006, Spain; josej.astrain@unavarra.es (J.J.A.); jesusv@unavarra.es (J.V.)

**Keywords:** judo, wireless sensor networks, ray launching, interference

## Abstract

In this work, the performance of ISM 2.4 GHz Wireless Sensor Networks (WSNs) deployed in judo training venues is analyzed. Judo is a very popular martial art, which is practiced by thousands of people not only at the competition level, but also as part of physical education programs at different school levels. There is a great variety of judo training venues, and each one has specific morphological aspects, making them unique scenarios in terms of radio propagation due to the presence of furniture, columns, equipment and the presence of human beings, which is a major issue as the person density within this kind of scenarios could be high. Another key aspect is the electromagnetic interference created by other wireless systems, such as WiFi or other WSNs, which make the radio planning a complex task in terms of coexistence. In order to analyze the impact of these features on the radio propagation and the performance of WSNs, an in-house developed 3D ray launching algorithm has been used. The obtained simulation results have been validated with a measurement campaign carried out in the sport facilities of the Public University of Navarre. The analysis is completed with the inclusion of an application designed to monitor biological constants of judokas, aimed to improve their training procedures. The application, that allows the simultaneous monitoring of multiple judokas (collective workouts) minimizing the efforts of the coach and medical supervisor, is based on commercial off-the-shelf products. The presented assessment of the presence of interfering wireless systems and the presence of human beings within judo training venues shows that an in-depth radio planning is required as these issues can have a great impact in the overall performance of a ISM 2.4 GHz WSN, affecting negatively the potential applications supported by wireless channel.

## 1. Introduction

Nowadays judo is a sport practiced by thousands of people around the world, being one of the most popular martial arts. In addition to the many worldwide competitions that are held under the auspices of the International Judo Federation, judo has been adopted in many countries as part of the physical education programs at different school levels. Thus, there is a huge variety of different training venues where judo is practiced. All these venues usually have some common aspects (such as having a tatami), but in general, each training venue has specific morphological aspects, making them unique complex indoor scenarios in terms of radio propagation due to the size of the venue itself and the presence of different furniture elements (e.g., cupboards, benches, chairs), columns, pads and other elements and equipment that can be found within training gyms, which will have a strong influence in the propagation of the electromagnetic waves as they generate phenomena such as reflection, refraction and diffraction. Therefore, the deployment of WSNs in such environments requires a previous radio planning work, especially considering that electromagnetic interferences created by other wireless sources such as personal portable devices and other wireless systems such as WiFi or other WSNs are likely to happen, making the coexistence in such indoor environments a complex task. Furthermore, in addition to the morphology of the training venues and the potential interferences, the presence of human beings within the scenario is a major issue in terms of radio propagation, especially taking into account that the person density in this kind of scenarios can be high as many judokas can be practicing and exercising at the same time.

Even though in the literature many wireless-based applications have been developed for different sport activities such as movement activity monitoring for sprinters [1], arm symmetry investigations for swimmers [2], tracking hip angles for cyclists [3], detection of illegal race walking [4], effort control systems [5] and general monitoring systems based on Ambient Intelligence and Internet of Things (IoT) [6,7,8], there are very few works related to the deployment of the WSNs within sport venues, and they show WSN deployments within sport venues much larger than judo training venues, such as stadiums [9,10]. When it comes to Wireless Body Area Networks (WBANs) and wearable devices related to sport activities, wearable sensors for training improvement, gait analysis, monitoring sport performance and coaching support have already been developed [11,12,13], but there are very few that deal with wireless-based applications in martial arts or contact sports [14,15,16]. Specifically related to judo, which is the sport under analysis in this work, some action recognition works have been published [16,17], but there is no reported work about radio planning analysis nor development of applications for the practice of judo except a radio propagation analysis paper written by the same authors of the present work [18]. Therefore, there is a neediness of in depth radio planning studies for judo training environments, especially taking into account the potential interferences that will be in such environments due to the development of Smart Cities and the IoT, where the radio planning will be a key issue in order to optimize the performance of WSN-based applications.

In this work, an in-house developed 3D ray launching algorithm has been employed in order to assess the impact that the presence of human beings has in the radio propagation within a real judo training environment, with the aim of developing potential applications based on wearable wireless devices. For that purpose, in Section 2 the 3D ray launching simulation tool and the description of the scenario under analysis are presented. In Section 3, firstly the validation of the simulation technique is presented. As ZigBee technology has been previously used for other sport monitoring systems [19,20], in this work ZigBee-compliant modules operating at ISM 2.4 GHz band have been used to take measurements within the real judo training scenario with the aim of analyzing the radio propagation as well as for validating the used simulation tool. Then, simulation results for cases with different number of human beings within the scenario are shown, which have been obtained with the inclusion of an in-house developed human body computational model [21]. Additionally, the radio planning analysis is completed with the interference level and Signal to Noise Ratio (SNR) estimations for an emitting WiFi hot spot placed within the scenario. In Section 4 a WiFi and ZigBee-based application for monitoring judokas’ biological constants during training sessions is presented. Finally, in Section 5 the obtained results are discussed.

## 2. Materials and Methods

### 2.1. The 3D Ray Launching Method

In complex indoor environments like judo training venues, where the morphology of the scenario and the presence of human beings greatly affect the electromagnetic wave propagation, it is a major issue to conduct a radio propagation analysis before the deploying of a WSN in order to obtain an optimized performance in terms of energy consumption and throughput. Traditionally, empirical methods such as Okumura Hata or COST-231, among others, have been used for that purpose. They provide very rapid results, but due mainly to the multipath propagation phenomenon that happens within complex indoor scenarios, these results are not accurate enough for the scenarios presented in this work, as it is shown later in this work. On the other hand, there are deterministic methods (e.g., Method of Moments and Finite Difference Time Domain), which provide very accurate results. They are based on the resolution of Maxwell’s equations, and the drawback is that they have a high computational cost, i.e., they are highly time-consuming, and therefore, they are used to simulate devices with its immediate surroundings, not a whole environment. As a midpoint, the ray launching and ray tracing methods offer a good trade-off between accuracy and calculation time, providing quite accurate results for a whole environment [22].

**Figure 1:**
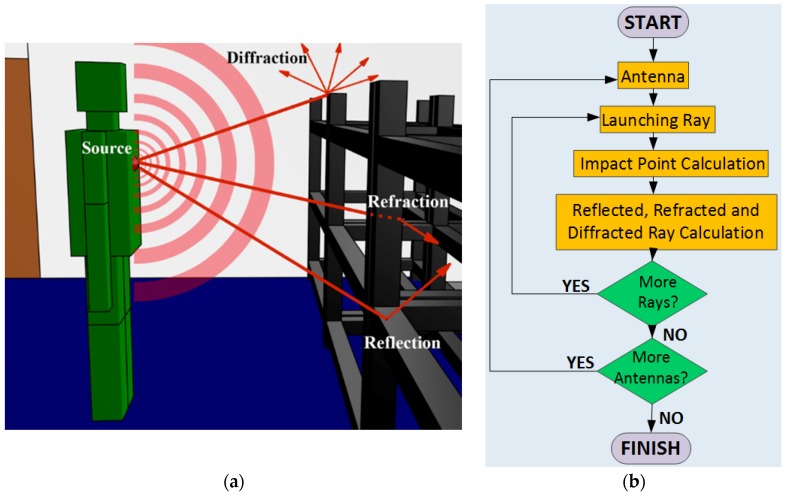
(**a**) Principle of operation of the in-house 3D ray launching method; and (**b**) functional diagram of the algorithm.

In this work, an in-house developed 3D ray launching simulation tool has been used in order to assess the impact that the morphology of the scenario and the presence of human beings have in the radio propagation at 2.4 GHz band within Judo training venues. The presented 3D ray launching algorithm is a deterministic method, based on geometrical optics and geometrical theory of diffraction. The principle of operation of the algorithm lies in the rays that are launched from a specified source in a solid angle (i.e., in 3D directions, both vertical and horizontal) with a predetermined angular separation. Before that, the entire volume of the scenario under analysis is divided into a parameterized mesh with different sizes of cuboids. These cuboids resolution is an input parameter that depends on the dimensions of the scenario. Then, the transmitter antenna is placed at a specific position within the scenario and rays are launched in a specified solid angle. The impact point with an obstacle or with a wall is calculated for each ray, calculating the new angles of the reflected, refracted and diffracted rays. When a ray impinges with a surface of an obstacle, new reflected and refracted rays are generated based on geometrical optics (GO), and when a ray impact with an edge, a new family of diffracted rays are created. These new diffracted rays are based of the geometrical theory of diffraction (GTD) and its extension the uniform theory of diffraction (UTD). While those rays are propagating in the space, all the parameters related with propagation are stored in each cuboid of the defined mesh, for later calculate different results such as received power, power delay profiles, delay spread or signal to noise ratio, among others. For a better comprehension, in Figure 1 this principle of operation is illustrated for a wearable transmitter, as well as the functional diagram of the algorithm. It is important to note that the properties of all the materials of the objects present within the scenario are considered at the frequency of operation (dielectric constant and conductivity). It is also important to mention that the ray launching simulations are mono-frequency, i.e., the results are obtained for a single frequency of operation. The detailed operating mode of the algorithm has been previously published [23], and it has been used and validated in different indoor environments [24,25,26], including judo environments [18].

**Figure 2:**
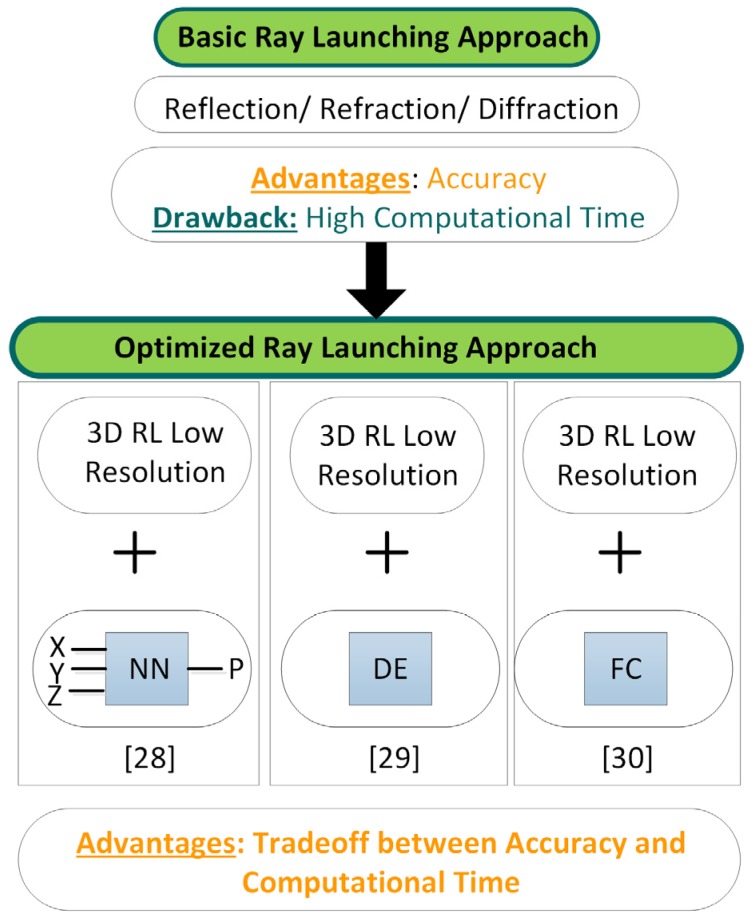
Performance of the in-house 3D ray launching approach.

For optimum performance of the algorithm, it is highly important to take into account different aspects such as the convergence analysis of the algorithm considering an adequate range for the input parameters. A convergence analysis of the algorithm can be found in [23,27]. These works present the parameter values to be considered in the ray launching approach for optimal performance in terms of accuracy and computational time. The analyzed parameters are the number of reflections, the angular resolution of the launching rays and the number of diffracted rays. It is also worth to mention that for large scenarios the computational time of simulations can be really high depending of the needed accuracy for the results. Because of that, novel hybrid techniques, combining the in-house ray launching approach with different techniques such as neural networks [28], the diffusion equation [29] or collaborative filtering [30] have been also analyzed. These techniques achieve accurate results with considerable lower computational times. The combination of these techniques with the ray launching approach gives an optimized ray launching methodology that is more robust for complex scenarios and it can be used for more applications (i.e., more complex judo training venues with a large number of judokas could be analyzed with such techniques). For better comprehension, Figure 2 shows a scheme of the improvement of the in-house ray launching technique, which have led to the optimal approach combining different techniques.

### 2.2. Scenario Under Analysis

**Figure 3:**
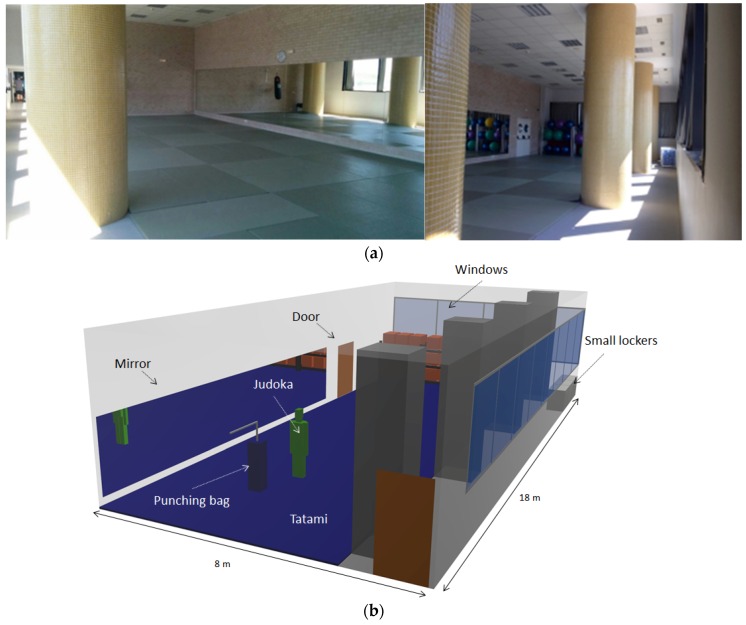
(**a**) Real scenario under analysis; (**b**) Schematic view of the created scenario for 3D ray launching simulations.

The scenario where the radio propagation analysis has been carried out is a gym located in the facilities of the Public University of Navarre. The dimensions of the scenario are 18 m (long) × 8 m (wide) × 4.8 m (height) and it contains a tatami of 17.5 m × 6 m, where judo is practiced. As happens in many judo training venues, other sports and physical activities are also practiced, therefore, the scenario contains typical elements easily found in many indoor training environments, such as a punching bags, big foam balls, doors, columns, a mirror and some furniture elements such as small lockers and shelves. All these elements make the scenario a complex indoor environment in terms of radio propagation, as the number of reflections, refractions and diffractions will be increased, making the multipath propagation the main phenomenon within the scenario, much more when persons are present. Figure 3 shows the real scenario and the schematic view of the scenario created for the 3D ray launching simulations.

Apart from the real size of the different elements of the scenario, the materials of all the objects within the scenario have been carefully chosen in order to obtain an electromagnetic behavior as closer as possible to the real materials. Table 1 shows the main materials used for the definition of the objects within the scenario, which both their relative permittivity and conductivity at the frequency of operation of the wireless system have been obtained from [31]. As the main objective of this work is to analyze the effect of the presence of persons on the radio wave propagation, the definition of the scenario has been completed with the inclusion of an in-house developed human body computational model [21], which is the most complex element within the scenario as the model has been built highly detailed with a broad range of different tissues within it and taking into account body parts such as skin, different organs, muscles and bones, all of them defined by their respective dielectric constant (i.e., relative permittivity) and conductivity values. Thus, in addition to analyzing the effect of the presence of people on the radio propagation, the inclusion of the human body model permits the propagation study of wearable devices, as it can be seen later in this work.

Once the scenario for the 3D ray launching has been created, the simulation parameters regarding the launching of rays and the wireless communication such as frequency of operation, antenna gain or cuboids size have to be defined. In Table 2 the main parameters are shown, which have been the same for all the simulations performed in this work. It is worth noting that the parameters have been chosen in order to match those of the real ZigBee-based devices and antennas used for the measurement campaign shown in next section.

## 3. Results

**Figure 4:**
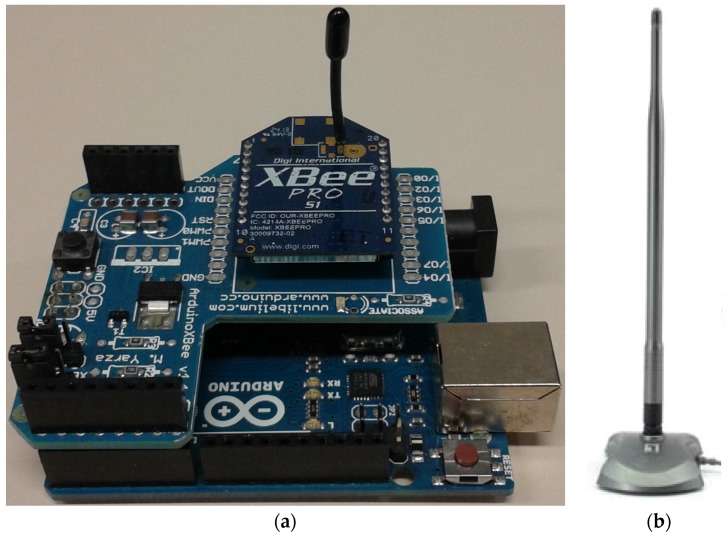
(**a**) XBee-Pro module used as transmitter; (**b**) 2.4–2.5 GHz band monopole antenna used as receiver.

**Figure 5:**
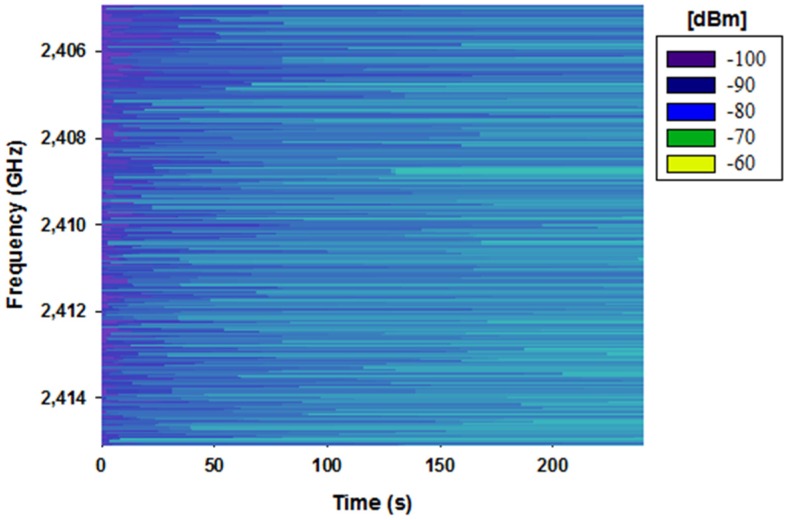
Measured spectrogram within the scenario under analysis for the ZigBee channel 12.

In this section, the simulation results for the assessment of the influence that the presence of human beings has on the radio propagation in judo training environments are shown. Firstly, the validation of the 3D ray launching method for its use in the presented scenario has been carried out. For that purpose, a measurement campaign within the real scenario has been made in order to compare these measurement results with those obtained by the 3D ray launching simulations. On one hand, a ZigBee-compliant XBee-Pro module with a 1.5dBi gain monopole antenna mounted on an Arduino board has been used as a transmitter (see Figure 4a). It has been connected to a laptop via USB cable and placed on a standing judoka’s chest, at 1.35 m height. The wireless mote has been configured to operate at 2.41 GHz, which corresponds to the channel 12 of the IEEE 802.15.4 standard. The transmitted power level has been set to 10 dBm and the bitrate to 250 Kbps, with the ‘Serial Interface Data Rate’ set to 125 Kbps (bauds per second). On the other hand, an OAN-1070 monopole 7 dBi gain antenna for ISM 2.4 GHz band operation connected to an Agilent FieldFox N9912A portable spectrum analyzer has been used as receiver. Figure 4 shows both the transmitter mote and the receiving antenna. Before the measurement campaign, a spectrogram has been measured in the center of the scenario under analysis in order to evaluate if any interference could affect the transmission over the chosen wireless channel. It has been measured with the antenna shown in Figure 4b connected to the Agilent FieldFox N9912A portable spectrum analyzer. Figure 5 illustrates the obtained spectrogram. As can be seen, there is not any signal interfering the wireless channel 12, which is centered at 2.41 GHz with a bandwidth of 3 MHz.

**Figure 6:**
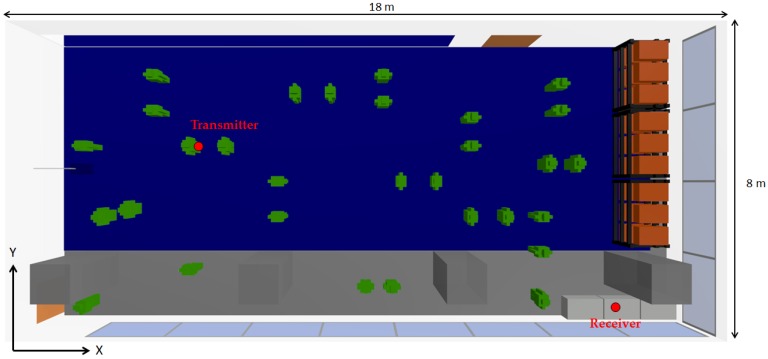
Upper view of the scenario presented in Figure 3 with the position of the transmitter element and the linear distances where the measurements have been taken (white dashed lines).

**Figure 7:**
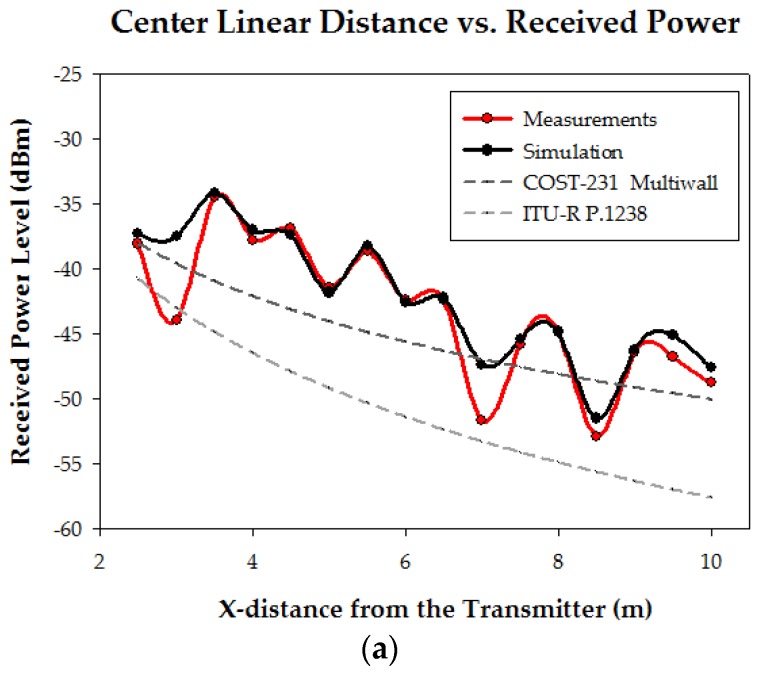

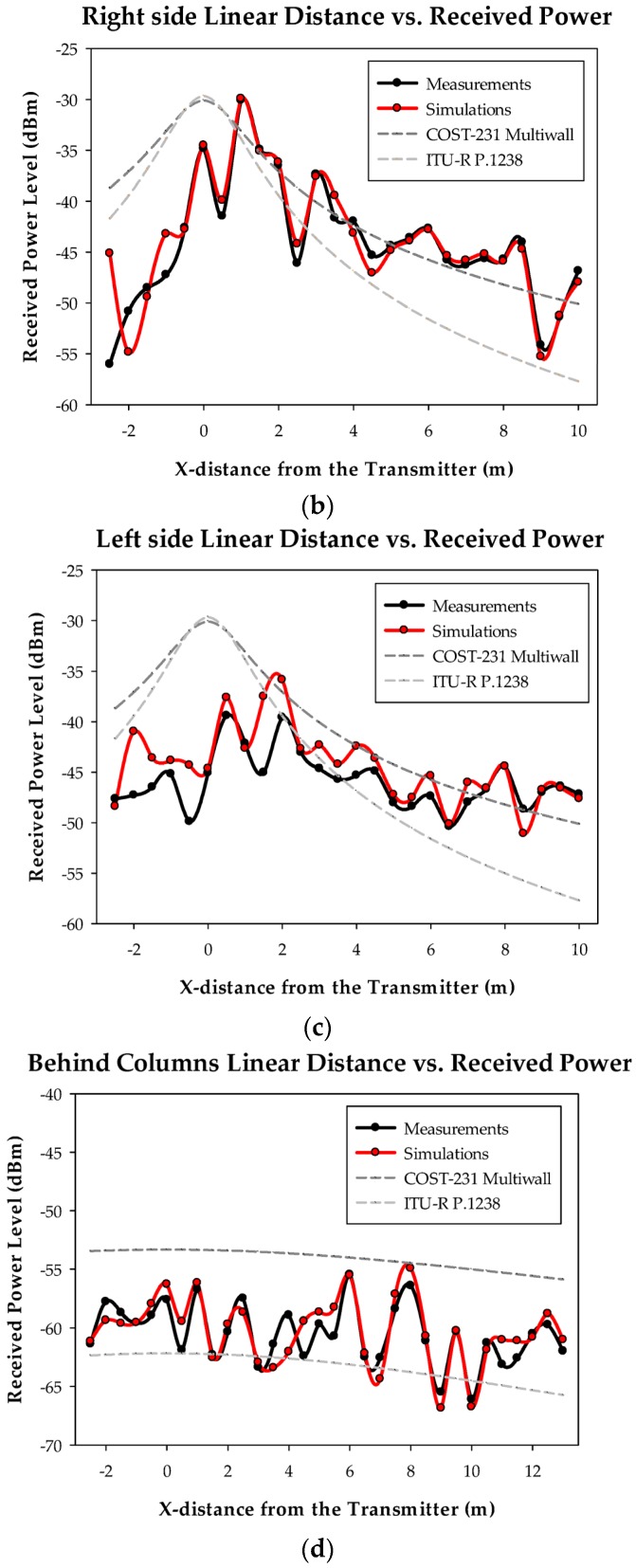
Linear distance vs. received power level corresponding to the dashed lines depicted in Figure 6: (**a**) Center; (**b**) Right side; (**c**) Left side; (**d**) Behind columns.

The configuration of the scenario for the radio propagation measurements is represented in Figure 6, where the standing judoka with the transmitter on the chest can be seen. The white dashed lines represent the linear distance where the measurements have been taken. The height is 1.35 m, the same height of the transmitter on the chest. The measured power level values are depicted in Figure 7, where the comparison with the estimations obtained by the 3D ray launching method is shown. Besides, estimations by traditional empirical models such as COST-231 and ITU-R P.1238 are included in the graph in order to compare them with the in-house 3D ray launching simulation tool. As can be clearly seen in Figure 7, the empirical models follow the tendency of the measurements curve, but they do not fit it properly due to the rapid variations of the received power, which is due to the multipath propagation. Besides, the empirical models do not take into account the effect of the presence of the judoka. Thus, the estimated power levels for x-axis negative values (i.e., behind the judoka) obtained by the Cost-231 Multiwall and ITU-R F.1236 empirical models are worse since the losses due to the human body are not taken into account (see Figure 7b,c). On the contrary, the 3D ray launching algorithm takes into account all the elements within the scenario, including the judoka, and its estimations fit very well the variations observed in measurements. In fact, taken into account the 100 measurement points represented in Figure 7, the mean error between measurements and 3D ray launching estimations is 0.76 dB, with a standard deviation of 2.07 dB. These values have been obtained by the following well known formulas:

(1) $$\mathrm{Mean}\;\mathrm{Error}=\bar{x}=\frac{\sum_{i=1}^{n}x_{i}}{n}$$

(2) $$\mathrm{Standard}\;\mathrm{Deviation}=\sqrt{\frac{\sum_{i=1}^{n}{\left(x_{i}-\bar{x}\right)}^{2}}{n}}$$

where *x_i_* is the error between each measurement and the simulation value and *n* the number of samples (100 measurements points). It is worth noting that the obtained low error is due to the methodology for obtaining the measurement values. Measuring the received power by a spectrum analyzer is much more accurate than the RSSI values provided by wireless transceivers, which in general give an error from 1 dB to up to 10 dB, depending on the hardware used. As an example, this effect is addressed in [32].

**Figure 8:**
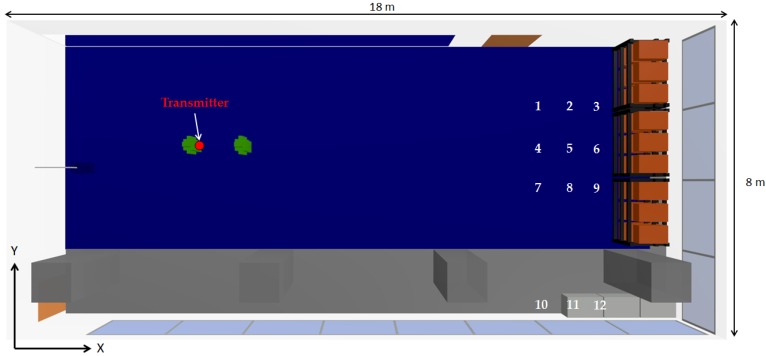
Upper view of the scenario with the new measurement configuration. The white numbers represent the measurement points.

**Figure 9:**
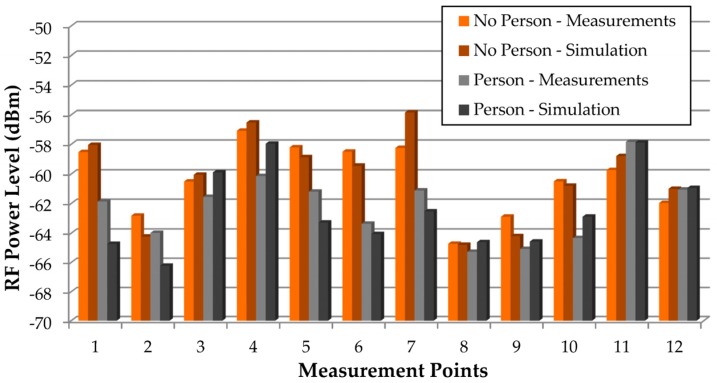
Measurements vs. 3D ray launching simulation results, both for the presence of an extra person and without the person. The measurement points correspond to those shown in Figure 8.

As the aim of this work is to assess the impact of the presence of persons within this kind of scenario, once the validity of the results obtained by means of the 3D ray launching algorithm for the scenario without people (except the judoka with the transmitter) has been made, new measurements and simulations with the inclusion of an extra human being (2 m in front of the judoka with the transmitter) have been performed in order to validate the inclusion of the in-house developed human body model. Figure 8 shows the position of the persons and the new measurement points, represented by white numbers (from 1 to 12). The measurement points are at the height of 1.35 m. Figure 9 shows the comparison between measurements and 3D ray launching simulations. In addition to the results with the inclusion of the person in front of the transmitter, the results without that person are also shown. Thus, besides the accuracy of the obtained estimations, the effect of introducing a person can be seen.

**Figure 10:**
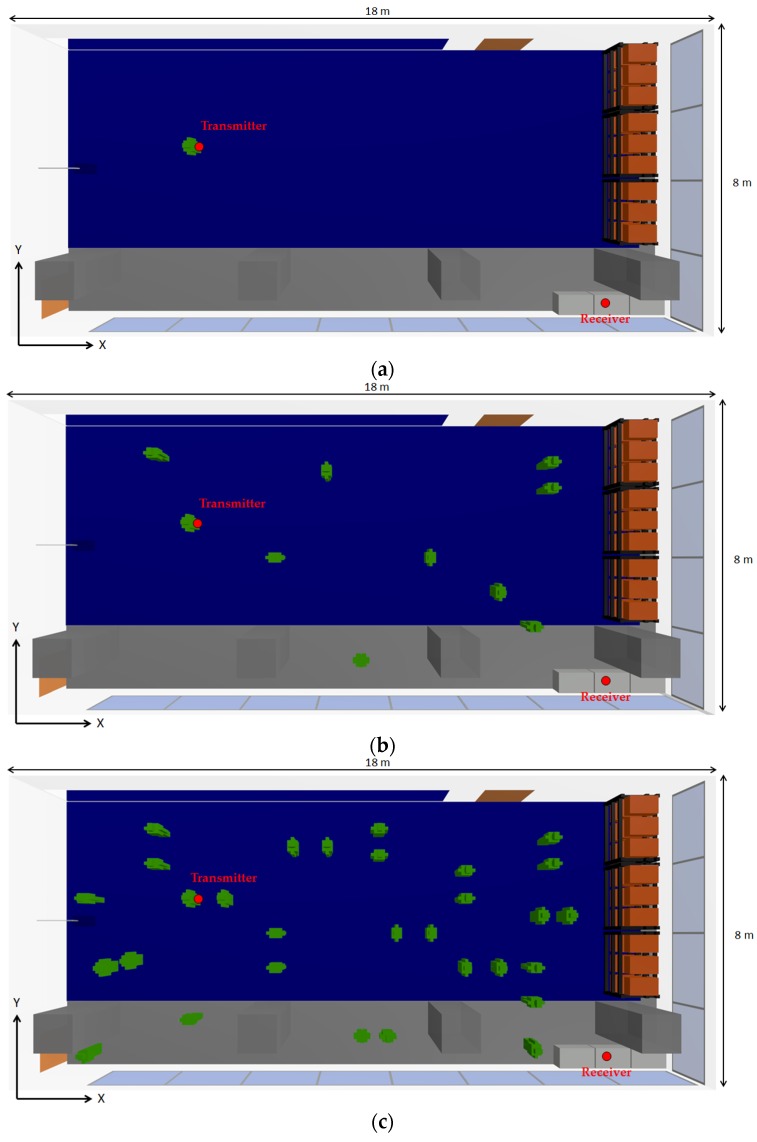
Upper view of the scenario under analysis with the position of the transmitter and the receiver devices (red dots) for the three cases of human being density: (**a**) Without persons; (**b**) With 10 persons; (**c**) With 30 persons.

As expected, the received power level is lower when the extra person is included in the scenario. It can be also seen that the accuracy of the estimations is also high when the human model is included in the simulated scenario. In order to gain insight on the assessment of the impact of the presence of persons within the scenario, further simulations have been performed. For that purpose, a XBee-Pro transmitter has been placed on the chest of a judoka (represented by a red dot in Figure 10), at 1.35 m height, and a receiver has been placed on the small lockers at height of 0.8 m (represented by a red dot in Figure 10), emulating a wireless link between a wearable wireless sensor and a laptop acting as a sink, receiving the information of the deployed WSN.

Three different cases have been simulated, maintaining both the transmitter and the receiver in the same place, but changing the person density within the scenario. The three cases are with the only presence of the person who has the transmitter, the presence of 10 persons and the presence of 30 persons. All simulations are static. The distribution of the persons throughout the scenario has been chosen randomly, but taken into account that some of them represent training Judokas (placed on the tatami) and others represent people outside the tatami. Figure 10 shows the upper view of the scenario with the three different simulated cases. The simulations with persons have been carried out with the aid of the previously mentioned in-house developed human body model.

**Figure 11:**
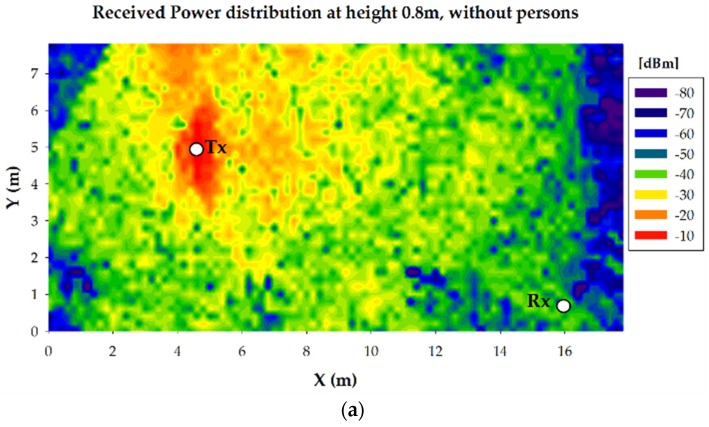

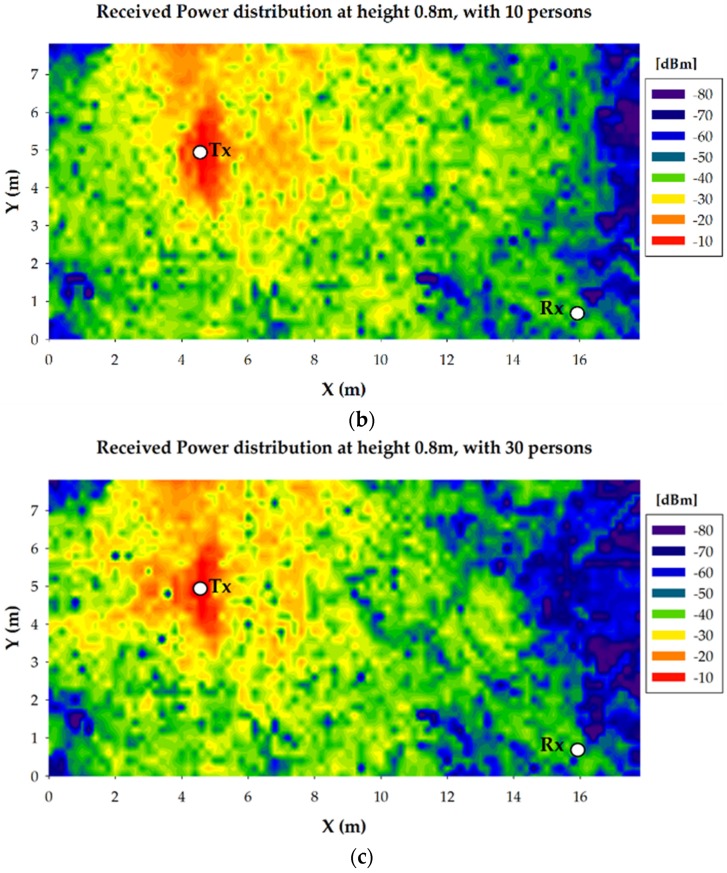
Estimated received power level distribution plane at height 0.8 m for the transmitter on the Judoka’s chest, (**a**) without more persons in the scenario; (**b**) with 10 persons and (**c**) with 30 persons.

**Figure 12:**
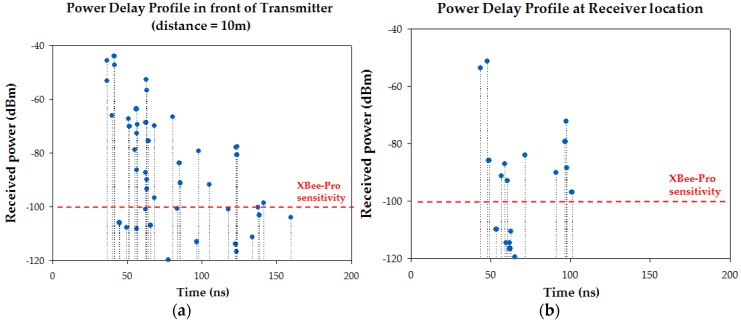
Power delay profiles at two different positions: (**a**) In front of the transmitter on the judoka’s chest at a distance of 10 m; (**b**) At the receiver location.

As it is previously stated, the parameters used for the 3D Ray Launching simulations have been chosen to match the characteristics of ZigBee-compliant XBee-Pro devices operating at ISM 2.4 GHz band. Table 2 shows them. Figure 11 shows the bidimensional plane of the estimated received power level at 0.8 m height (i.e., the height of the receiver) for the three cases under analysis. Both the position of the transmitter and the receiver are represented by white dots with the text ‘TX’ and ‘RX’ respectively. Note that the position of the transmitter has been drawn although its real position is at height 1.35 m. The short term variation of the received power due to multipath propagation seen in Figure 7 can be also seen throughout the scenario. As the multipath propagation has a strong impact on the received power level, even more in the zones where the surrounding obstacles affect the LoS, two power delay profiles (PDP) are presented in Figure 12 for the case without persons in order to gain insight in this matter. Specifically, one PDP corresponds to a point just in front of the transmitter on the Judoka’s chest at a distance of 10 m. The second one corresponds to the point where the receiver is placed (see Figure 10). It can be clearly seen how the first components arrive earlier to the position in front of the transmitter due to the LoS and the shorter distance. Besides, less multipath components and with lower power level arrive to the receiver location, as it is expected due to the surrounding obstacles. In addition, a dashed red line is depicted to show which components have a power level lower than the sensitivity of the used XBee-Pro modules.

**Figure 13:**
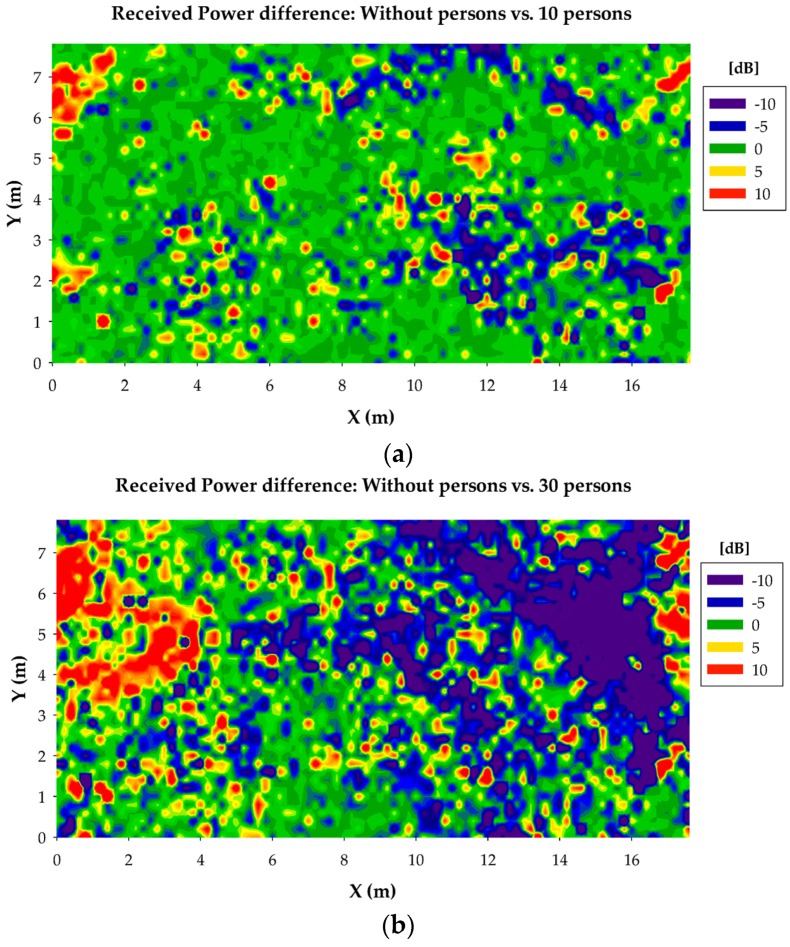
Received power level difference for the bidimensional plane at 0.8 m height between the results without persons and (**a**) with 10 persons; (**b**) with 30 persons.

Regarding the effect of the presence of persons, the difference between the case without persons and with 30 persons can be clearly seen, as the received power decreases at higher distances. On the contrary, the difference between the case without persons and with 10 persons is hard to note with a naked eye. In order to gain insight in the effect of the presence of persons, in Figure 13 the difference between the received power plane for the case without persons and the cases with persons is represented. As expected, the difference for the case of 30 persons is much bigger than for the case of 10 persons. Even so, the differences for the case of 10 persons are significant in terms of wireless channel quality for many points in the scenario, as the decrement of few dB in the received power can lead to a failure in the wireless communication due to either a received power level lower than the sensibility of the receiver device or an insufficient SNR value.

**Figure 14:**
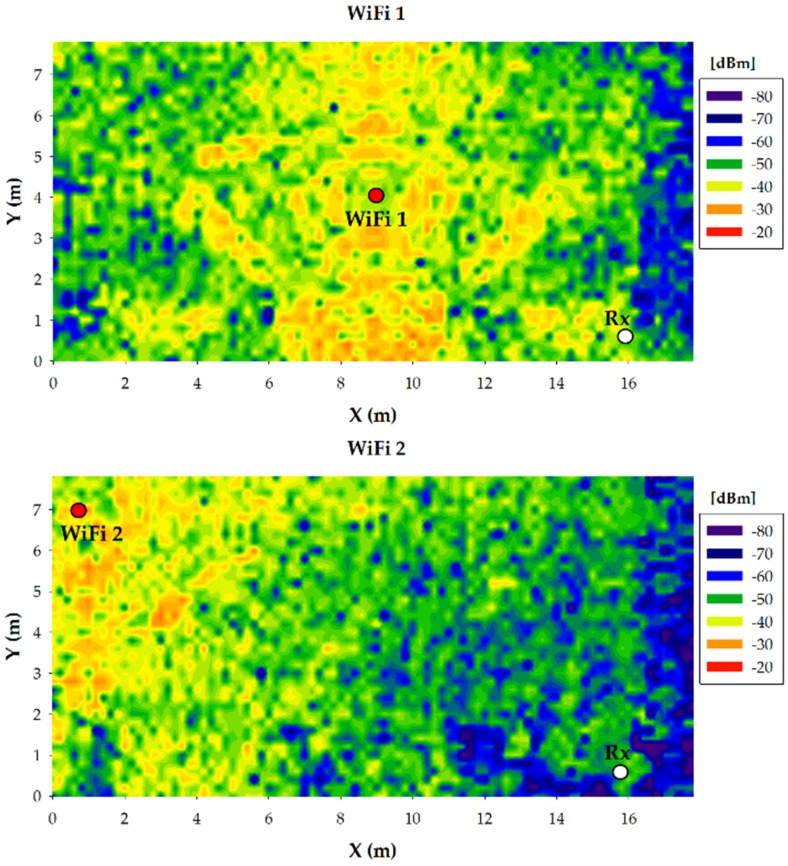

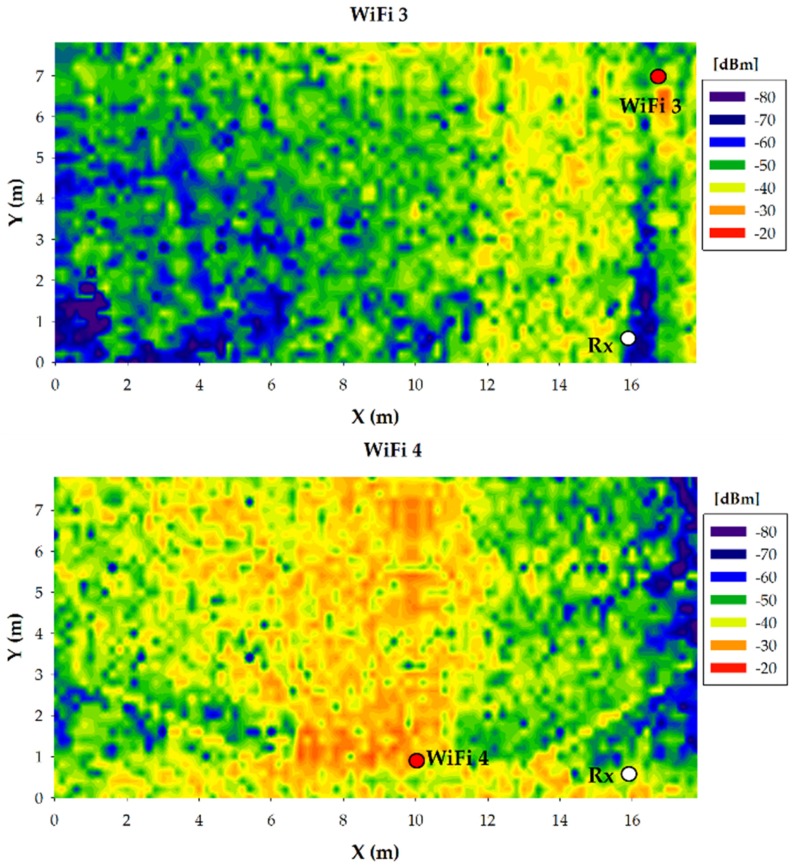
WiFi power distribution for the bidimensional plane at 0.8 m height for the case without persons. The red dots represent the WiFi access point locations.

**Figure 15:**
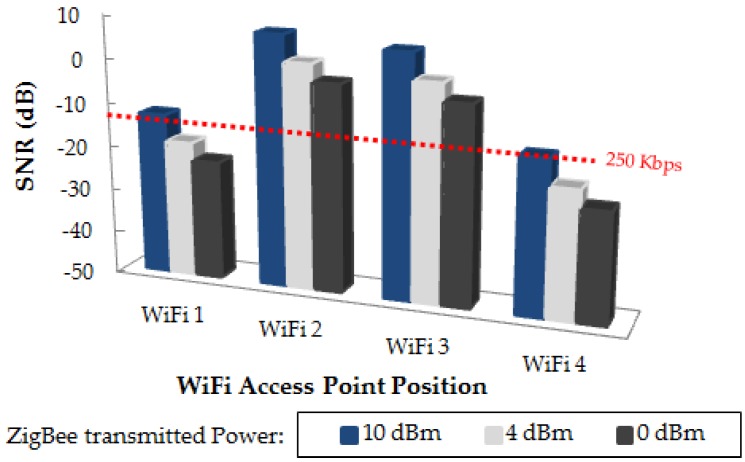
Estimated SNR values at receiver position for different WiFi access point positions, for different ZigBee transmitted power levels.

Once the effect of the presence of persons within the scenario under analysis in terms of received power level has been performed, how these results can affect the deployment of wireless transceivers in terms of data rate and energy consumption is presented. Firstly, the estimated received power values give the information needed in order to know if a specific wireless transceiver will receive the required minimum signal power to have a successful communication with the transmitter. This minimum signal power is given by the sensitivity of the transceivers. In this case, the XBee-Pro modules have a sensitivity of −100 dBm, which is surpassed by the received power for almost all the points throughout the whole scenario. But this is not enough to have a successful wireless communication between the transmitter and the potential receiver, as electromagnetic interference is likely to be present in such a scenario, even more in a future context aware scenario framed by the IoT and Smart City environments. In order to show the impact that interferences could have on the wireless communication within judo training environments, as an example, four WiFi access points have been placed in the scenario, fixed to the ceiling (height of 3.9 m), emitting 20 dBm at the same frequency of operation of the ZigBee motes. The ZigBee transmitted power level has been set to 10 dBm. The location of the WiFi access points as well as the estimated WiFi power distribution at the height of 0.8 m for each access point is presented in Figure 14, for the case without persons. In order to assess if the ZigBee communication can be successfully achieved when those WiFi access points are transmitting, the relation between the received ZigBee signal power and the interference produced by the WiFi access points has been calculated, i.e., the SNR. Note that both the WiFi access points and the ZigBee motes usually transmit traffic burst, not continuously. Therefore, the interference between those two wireless systems happens when both systems transmit at the same time, i.e., when collision of both signals happen. The SNR has been calculated by the following well known formula:

C = BW × log_2_(1 + S/N) (3)

where C is the channel capacity in bps (250 Kbps fixed for ZigBee), BW is the communication system bandwidth in Hz (3 MHz for ZigBee) and S and N are the power levels of the received signal and noise respectively (in Watts). The required minimum SNR for a successful ZigBee communication is −12.26 dB. Table 3 shows the simulation results of the received ZigBee signal power level as well as WiFi interference levels at the receiver location (Rx), and in Figure 15 the estimated SNR values at receiver position for the different WiFi access point positions are depicted. The dashed red line represents the previously calculated minimum SNR value of −12.26 dB. As can be seen, the potential positions of the wireless transceivers, both the motes of our network and the interfering devices, have a great impact on the performance, which at the same time will depend strongly on the morphology of the scenario.

**Figure 16:**
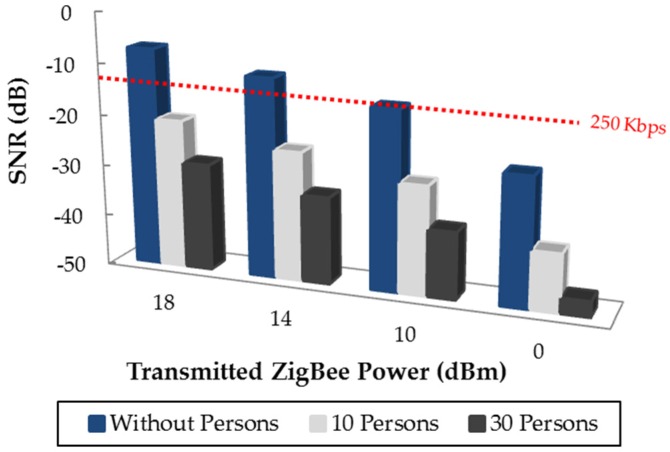
Estimated SNR values for different configurations of the Tx-Rx wireless link when the WiFi 4 access point is interfering.

In order to gain insight in how the presence of human beings affects the performance of a ZigBee communication in terms of SNR, the WiFi 4 position has been taken as an example and in Figure 16 the SNR calculated at the receiver position (Rx) for the three cases without persons, with 10 persons and with 30 persons is presented. The x-axis indicates different transmission power levels for the ZigBee sensor of the judoka’s chest. Note that although the European regulations allow transmitting up to 10 dBm, the inclusion of higher transmitting power levels in the analysis is due to the limitations are different in some other parts of the world and there are commercial devices which can transmit higher power level (e.g., the XBee-Pro modules used in this work, which transmit up to 18 dBm). Table 4 shows the simulation results of the received signal power levels as well as WiFi interference levels at the receiver location (Rx) when ZigBee transmits 10 dBm. As can be seen, for the WiFi 4 position, the received WiFi signal is not affected by the inclusion of persons, which is mainly due to the shorter distance between the WiFi access point and the receiver. But the received ZigBee signal is affected significantly, which is expected as in the case under analysis the included persons are in the path between the ZigBee transmitter and the receiver. Back to Figure 16, the difference between the cases without persons and including persons is due mainly to the lower ZigBee signal power received at the Rx point, which lead to a lower SNR value. As expected, the SNR increases when the transmitting power levels are higher. But it is worth noting that the 250 Kbps data rate is not achievable transmitting 10 dBm or less when collision between the WiFi and ZigBee happens. Increasing the transmitting power level above 10 dBm will avoid that problem, but at the expense of a higher energy consumption of the transmitter, which is likely to be powered by batteries. Instead, the proposed method based on the 3D ray launching algorithm can aid in finding an optimized WSN deployment, in order to obtain optimized transceiver placement in terms of achievable data rate as well as energy consumption.

Other potential communication systems could be employed in order to provide the required connectivity, such as WLAN or mobile communication systems. In general terms, coverage/capacity relations for low bit rate transmission (i.e., in the same range as 250 Kbps) would hold, given the fact that transmission power can be higher in both cases. Eventually, new coverage analysis would have to be carried out to analyze if quality of service parameters hold as a function of increased bit rate demand, a situation that could happen in services such as real time video are required. This is a topic for future work, as a function of required end-user application demands.

## 4. Judoka Monitoring Application

In [18] we developed an application aimed at monitoring the practice of judo focusing on facilitating the tasks of judo arbitration for the referee and to the two corner judges, helping the audience at competitions to understand the fighting and the scores given by the referees, and allowing the refinement of judo waza. Following this line of work, we have developed an application that monitors certain biological parameters of the judokas and oversees their efforts at training and competition, in order to improve the quality of training, while ensuring that there is not conducted un-recommended overexertion. The developed application uses commercial off-the-shelf (COTS) products. It has been designed to use standard manufactured products rather than customized, or bespoke, products. We have focused on minimizing the cost of the hardware equipment, to grant an easy integration of the different commercial equipment, and on developing a software that takes full advantage of the system with lowest energy consumption. The system integrates standard and well known tools.

Judo requires, as do other sports, the combination of a good physical preparation of both anaerobic and aerobic type, since a high resistance to withstand the duration of a bout as well as explosive and high-speed actions is required. When properly performed, Judo training can provide significant functional benefits and improvement in overall health and technique. We can distinguish three different phases needed to produce the big performance desired by a judoka when planning his/her training: the preparatory, competitive and transit periods. During the preparatory period, strength, speed, endurance, agility, flexibility, mobility and technical issues are addressed. The competitive period is devoted to increase the intensity of the activity, while the transit period is devoted to the functional regeneration of the judoka. In order to facilitate the collection of information that enables better planning and executing of training we have developed the application described below. The technical novelty of the proposal is the simultaneous monitoring of multiple judokas. When workouts are collective, the difficulty of monitoring the training conditions of all judokas makes it impractical for just a person, or for a reduced number of them, to supervise the activity of multiple judokas at a same time. For this reason it is necessary to propose a system architecture that grants those objectives are met.

**Figure 17:**
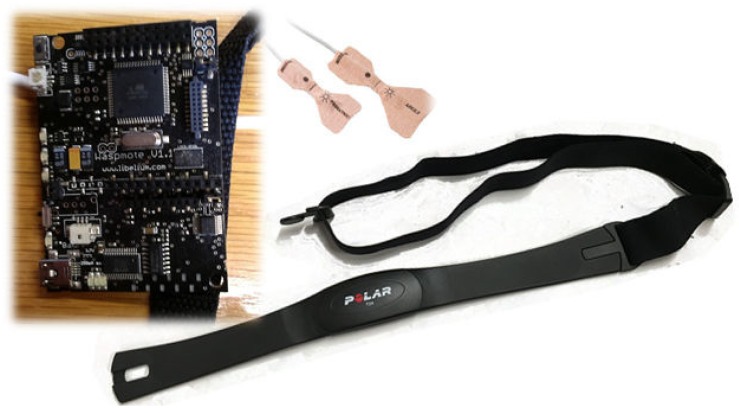
Hardware devices involved in the monitoring process.

**Figure 18:**
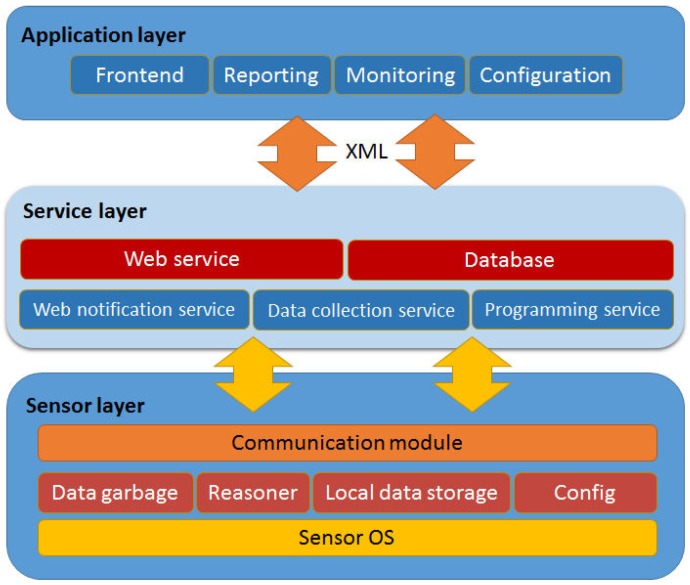
Software architecture of the monitoring system.

A noninvasive method has been followed for monitoring the judoka’s oxygen saturation (SO_2_) by reading his/her peripheral oxygen saturation (SpO_2_), while his/her heart rate is also measured. Figure 17 shows the devices involved in the sensing process, while Figure 18 describes the software architecture of the system, which consists of three layers: sensor, service and application layers. The device in charge of data sensing is a Waspmote node [33], which aggregates the data collected by a SpO_2_ sensor and a heart rate monitor and summits it to a web service in charge of permanent data storing. The sensor layer is located at the Waspmote node and includes a communication module, which allows data collection from sensor devices by means of a Bluetooth connection and WiFi communication with the service layer. The node provides a certain storage capacity, which is provided by an SD card. The node can be programmed by a small configuration module (Config) to interrogate the available sensors and acquire the corresponding information. This information is stored in the SD card through the local data storage module, and analyzed by a reasoner. The reasoner is responsible of discerning whether the values sensed suggest that a judoka is reaching a worrying level, and if so, it urgently notifies this fact to the service layer. The setup of the nodes establishes the refresh rate of the sensors. Internally, the node acquires information continuously from sensors, since it performs a continuous loop data refresh (data garbage). Data is always stored locally into the SD card and transmitted to the storage web service according to the network availability. If the reasoner does not observe any significant abnormality after evaluating each sensed data, it requests the delayed persistent storage of the data to the storage web service. The delayed transmission allows a better use of the available bandwidth, minimizing the transmission time in order to avoid collisions between bursts of messages. The node aggregates the information available and looks forward periods of low network activity to send the information to the correspondent web service. If the reasoner does not observe any significant abnormality after evaluating the data obtained, it requests the persistent storage of the data. If the reasoner appreciates any anomaly, it urgently informs this fact to the web notification service located at the service layer in order to take the required actions such as stopping the training session. Periodically, where this refresh rate can be modified by users when needed (Config module), the node sends the data aggregated during the last sensing period to the data collection service located at the sensor layer. This service stores the data collected into a relational database, in our case, we use a MySQL database server. All the modules of the sensor layer are implemented as pieces of code embedded in the cyclic algorithm executed by the node over its operating system.

**Figure 19:**
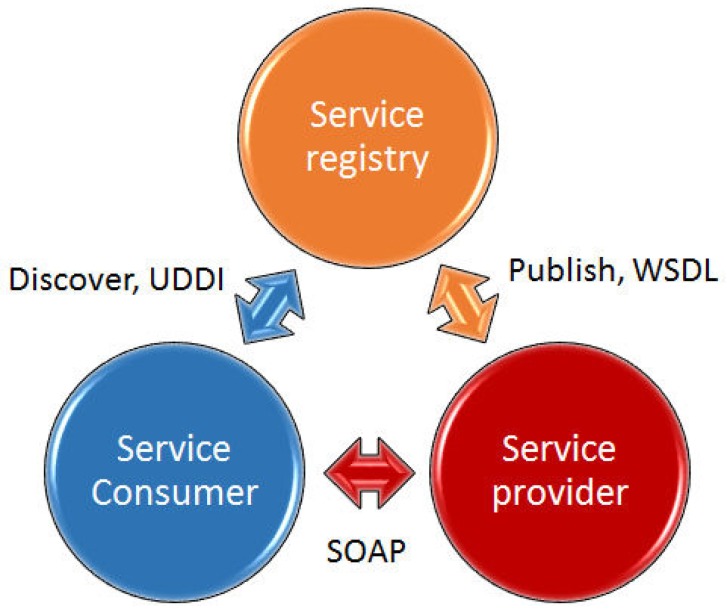
Web service architecture.

**Figure 20:**
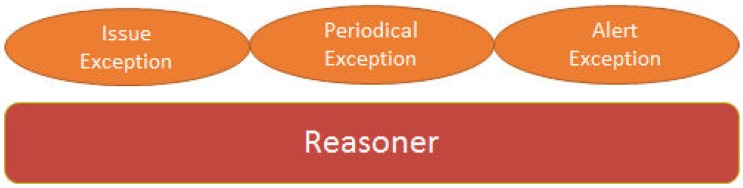
Possible issue notifications by the reasoner.

The service layer implements all its functionalities by means of web services. The three services provided (programming, data collection and notification) are web services. The programming services allow an easy and quick configuration of the sensors and their associated parameters. Thus, the user can determine through a web service what sensors correspond to what judoka, how often to monitor the acquired data, which alert thresholds should the reasoner take into account, and many others. Note that the node is cyclically executing that algorithm forever. The data collection service stores the received data into a database and acknowledges the storage to the sensor layer. This ensures that no information is lost, since the storage module periodically requests to the storage service the storage of all data pending. If a failure occurs when invoking the web service, the sensor will re-request the storage in the next iteration and will not give up until it reaches its target and the proper storage of information into the database is acknowledged. The notification service is in charge of alert notification from sensors (reasoner) to the application layer, although the information is also transferred to the storage service for its persistent storage. Those services, as well as the MySQL 5.6 and Tomcat 6 servers, are located at a laptop and communicate through a WiFi LAN network. The location of the servers in a laptop is due to mobility and portability reasons. We have tried to make the system the most portable possible. In Figure 19 the well-known architecture followed by the three services described above and the elements used to discover (UDDI), publish (WSDL) and invoke (SOAP) web services are depicted. Figure 20 shows the exceptions that can be thrown by the reasoner to the notification service. A *Periodical Exception* just implies a new storage iteration, while an *Issue Exception* implies a certain problem such as the notification of any previous communication problem, the storage request of pending data from previous failed storage cycles, etc.

Finally, the *Alert Exception* implies the urgent notification to the application layer of a serious incident such as the total or partial loss of connectivity, or something more important, such as any of the judokas has exceeded any of the risk thresholds previously established. In all cases, and according to the priority levels defined (alert, issue and period) the service layer notifies these exceptions to the application layer. Much of the potential traffic is minimized due to the pre-processing performed on sensors. The carried traffic is reduced, which prevents flooding and minimizes channel collision probability. In a collective training, in which several judokas compete simultaneously, multiple bursts of traffic between the sensors and the rest of the system must be considered. In addition, since a judoka competes against another, we frequently observe that both sensors try to communicate at the same time with the system, as both competitors simultaneously perform high-intensity efforts. For such reason, and to minimize the workload of the monitor, the filtering task performed by the reasoner is so relevant.

**Figure 21:**
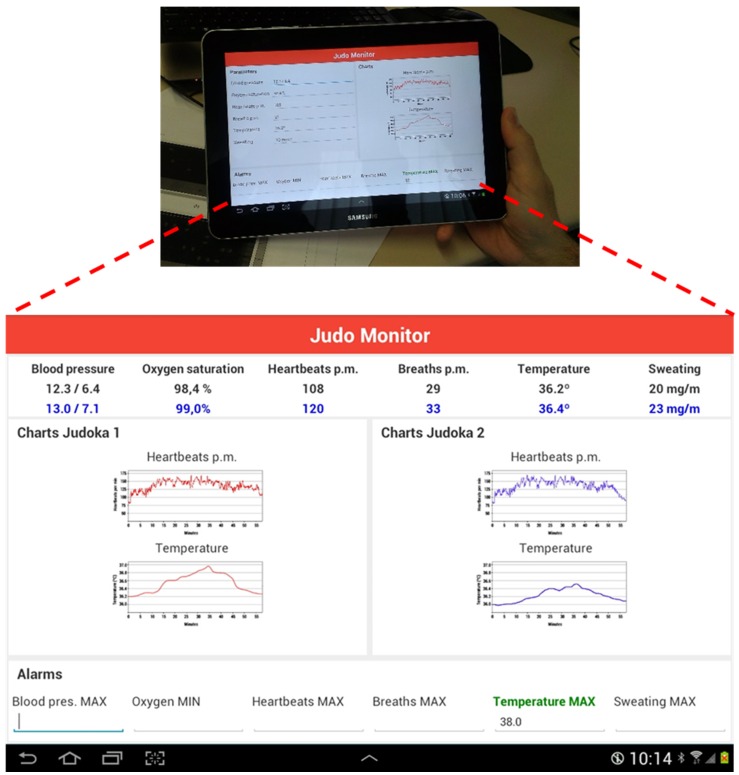
View of the presented monitoring app running in a tablet. The single (on the tablet’s screen) and dual (zoomed) monitoring of a randori are represented.

Finally, the application layer, which is implemented following the Model, View, Controller (MVC) pattern, mainly concerns four issues: frontend, reporting, monitoring and configuring. Following that scheme, an Android-based app has been developed, which is depicted in Figure 21.

The aim of the application is to monitor different biological parameters of judokas during their training exercises, such as randori (i.e., combat). The application allows the monitoring of a single judoka or a set of them. It also allows the simultaneous comparison of two judokas, usually those judokas who are fighting against each other. The user can select the judoka to be monitored from among all those available. While this monitoring is performed in real time, the application also allows re-displaying previous data already stored in the database. The frontend module is in charge of user login, data connection, user registration, the election of the judoka/s to be monitored and the customization of the graphic user interface. The monitoring module, depicted in Figure 21, is in charge of data comparison, alert notification to users and the real-time presentation of the data collected by sensors. The configuration module allows the setup of sensors, the enabling/disabling of sensors, the registration of new devices in the system, the publishing of new services, etc. The reporting module makes use of the Jasper Reports tool to provide powerful and useful reports aimed at improving the training methodology of judokas. The reporting module makes use of the Edrawsovt 2015 v7.9 tool to provide powerful and useful reports aimed at improving the training methodology of judokas. Reports provided aid to supervise and improve the training process. The user can select the type and number of indicators and graphs, allowing the customization of the reports provided. All the information gathered along the time is achieved into a database, so the system allows a wide number of individual and collective comparatives studies according to the weight divisions, the gender, category (junior, senior…), etc.

**Figure 22:**
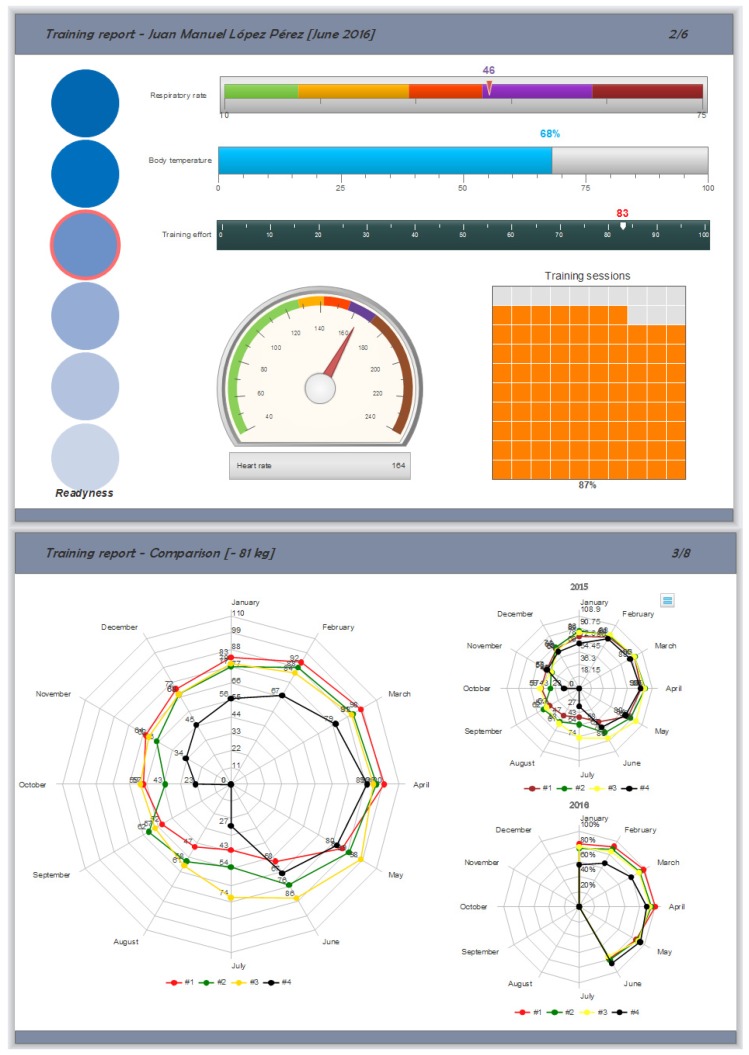
Reporting examples: individual report (**top**) and collective report by category (**bottom**).

Figure 22 illustrates some of the reports provided. The top figure shows the evolution of the training process of a certain judoka while the bottom part shows the evolution comparison among the four judokas belonging to the category of less than 81 Kg. As it can be observed in Figure 22 (bottom), judoka #4 has been injured for more than three months. One can note that the highest degree of training/competitiveness corresponds to the celebration of the European Judo Championship 2016 celebrated in Kazan (21 April, Russia). Right now, all the reports are generated on demand and stored into a private repository, while our future works focus on the use of a business intelligence tool as Pentaho to design and provide interactive dashboards.

## 5. Conclusions

In this work, the influence of the presence of human beings on the performance of WSNs at ISM 2.4 GHz band in judo training venues is analyzed by means of an in-house developed 3D ray launching algorithm, with the aid of an in-house developed human body computational model. The obtained results show the typical short term variations of the received power level due to the multipath propagation, which is usually the strongest propagation phenomenon in this kind of indoor environments. The comparison between simulation results and measurements show that the in-house developed 3D ray launching method is an accurate tool in order to obtain received power estimations within judo training environments, as the estimations fit the short term variations, in contrast to traditional empirical methods, which only give the tendency of the values.

The presented results show that in addition to the placement of the wireless transceivers (transmitters as well as receivers), which has a great impact in the power distribution throughout the scenario, the presence of persons is a key issue that has to be taken into account in order to obtain an optimized deployment of a ZigBee WSN in judo training environments. In fact, the high density of persons that can be found in this kind of scenarios can be a determining factor in the performance of the deployed ZigBee-based WSN in terms of achievable data rate and energy consumption.

The presented method can aid in obtaining the optimal wireless network deployment, configuration and performance, making the use of WSNs attractive for the adoption of applications within judo training venues, such as the biological constant monitoring application presented in this work. This method can be transferred for similar assessments of any judo training venue as well as other sport venues with similar morphological characteristics, where besides judo, other different sports could be practiced, such as other martial arts and diverse physical activities (e.g., yoga, gymnastics for seniors, any kind of dance or aerobics, just to name a few). As future work, the effect of the dynamic presence of human beings within this kind of scenarios as well as the analysis of other wireless technologies and operating frequencies will be interesting issues to analyze. Regarding the developed application, the software architecture proposed allows the implementation of new applications using other technologies or platforms (HTM5, IOS ...) with the least effort. Future works concern the integration of two tools as Weka and Pentaho in order to better define the rules of the reasoner and provide precise dashboards, respectively.

## Figures and Tables

**Table 1:** Material properties defined for the 3D ray launching simulations. Data obtained from [31].

Material	*ε_r_*	Conductivity (S/m)
Tatami	3	0.2
Concrete	25	0.02
Aluminum	2.2	37.8 × 10^6^
Polypropylene	3	0.11
Foam	1.4	0.021
Brick	4.44	0.11
Door	5.84	0.06
Glass	6.06	10^−12^
Plasterboard	2.02	0

**Table 2:** 3D ray launching simulation parameters.

Parameter	Value
Frequency	2.41 GHz
Transmitted Power Level	10 dBm
Transmitter/Reciever Antenna type	Monopole
Transmitter Antenna Gain	1.5 dBi
Reciver Antenna Gain	7 dBi
Launched Ray Resolution (Horizontal and Vertical)	1°
Permitted Maximum Reflections	5
Cuboids size	10 × 10 × 10 cm

**Table 3:** Simulation results at receiver point (Rx) for ZigBee transmitting 10 dBm and WiFi transmitting 20 dBm.

WiFi Location	ZigBee Signal Power (dBm)	WiFi Interference Power (dBm)
WiFi 1	−64.12	−51.16
WiFi 2	−64.12	−71.11
WiFi 3	−64.12	−68.99
WiFi 4	−64.12	−48.91

**Table 4:** Simulation results at receiver point (Rx) for ZigBee transmitting 10 dBm and WiFi transmitting 20 dBm.

Human Density Case	ZigBee Signal Power (dBm)	WiFi Interference Power (dBm)
Without Persons	−64.12	−48.91
10 Persons	−77.85	−48.91
30 Persons	−86.08	−49.02

## Abbreviations

The following abbreviations are used in this manuscript:

WSN	Wireless Sensor Network
SNR	Signal to Noise Ratio
ISM	Industrial, Scientific and Medical
IoT	Internet of Things
